# Characterization of Silicate Glass/Mullite Composites Based on Coal Fly Ash Cenospheres as Effective Gas Separation Membranes

**DOI:** 10.3390/ma16216913

**Published:** 2023-10-27

**Authors:** Elena V. Fomenko, Elena S. Rogovenko, Natalia N. Anshits, Leonid A. Solovyov, Alexander G. Anshits

**Affiliations:** Institute of Chemistry and Chemical Technology, Federal Research Center “Krasnoyarsk Science Center of Siberian Branch of the Russian Academy of Sciences”, Akademgorodok 50/24, 660036 Krasnoyarsk, Russia; rogovenko_elena1989@mail.ru (E.S.R.); anshitsnn@mail.ru (N.N.A.); leosol@icct.ru (L.A.S.); anshits@icct.ru (A.G.A.)

**Keywords:** coal fly ash, cenospheres, silicate glass, mullite, membrane separation, gas permeation properties, helium, neon

## Abstract

Membrane technology is a promising method for gas separation. Due to its low energy consumption, environmental safety, and ease of operation, membrane separation has a distinct advantage over the cryogenic distillation conventionally used to capture light inert gases. For efficient gas recovery and purification, membrane materials should be highly selective, highly permeable, thermally stable, and low-cost. Currently, many studies are focused on the development of high-tech materials with specific properties using industrial waste. One of the promising waste products that can be recycled into membrane materials with improved microstructure is cenospheres—hollow aluminosilicate spherical particles that are formed in fly ash from coal combustion during power generation. For this purpose, based on narrow fractions of fly ash cenospheres containing single-ring and network structure globules, silicate glass/mullite composites were prepared, characterized, and tested for helium–neon mixture separation. The results indicate that the fragmented structure of the cenosphere shells with areas enriched in SiO_2_ without modifier oxides, formed due to the crystallization of defective phases of mullite, quartz, cristobalite, and anorthite, significantly facilitates the gas transport process. The permeability coefficients He and Ne exceed similar values for silicate glasses; the selectivity corresponds to a high level even at a high temperature: αHe/Ne—22 and 174 at 280 °C.

## 1. Introduction

Currently, recycling industrial waste to produce high-tech materials with specific properties attracts great attention. Coal fly ash, the major byproduct (60–95%) from pulverized coal combustion [1,2], contains hollow aluminosilicate spherical particles, cenospheres, which can be useful in many industrial applications due to their unique properties, such as low bulk and apparent density, high thermal resistance, chemical stability, compressive strength, and low thermal conductivity [3,4,5,6]. Of the promising directions for the use of cenospheres that have been considered recently, the following can be noted: low-density ceramic [7,8] and metal syntactic foams [9,10], porous mullite ceramics [11], polymer–matrix composites [12], catalysts and carriers [13,14], composite materials for biomedical applications [15], and lightweight cement concrete [16].

The potential of the application of fly ash cenospheres for the production of functional and composite materials is associated with the possibility to stabilize their composition and properties as a consequence of various physical characteristics of individual globules (density, size, and magnetic properties). Increasing attention being is paid to the separation of cenosphere concentrates into narrow fractions with specific physicochemical properties; the systematic study of their composition and structure, including individual globules; and the identification of promising areas for further application [17,18,19]. The criterion for the suitability of cenosphere fractions in each specific case is the fulfillment of stringent requirements for the composition and structure of the glass–crystalline shell of globules. On the basis of well-characterized narrow fractions of cenospheres due to a definite composition and morphology of globules, new functional materials with predictable and reproducible properties have been obtained. Among such materials are highly selective membranes for helium capture [20,21].

The global cenospheres market is predicted to rise by an average of 11.9% by 2029 [22], which will allow the development of new areas for their application, one of which is the production of membrane materials. Membrane technology is a simple and efficient gas separation method with less energy consumption than traditional processes, such as pressure swing adsorption or liquefaction through compression and cooling. The separation and purification of light inert gases, helium and neon, play an important role in modern science and technology [23]. However, existing membrane materials (polymers, MOFs, zeolites, etc.) have an extremely low selectivity for He-Ne mixtures; for example, the most selective of them is polysulfone, which has an αHe/Ne value of no more than 5 [24].

At present, membrane gas separation is entering the stage of the directional design of new effective, highly selective, and highly permeable membrane materials characterized by an improved microstructure, chemical resistance, strength, and stability at elevated temperatures. Intensive research aims to develop carbon-based [25,26], organic-framework [27], mixed-matrix [28,29], and inorganic membranes [30,31].

A promising material for creating highly selective gas separation membranes is nonporous silicate glass [32,33]. Even at high temperatures, the αHe/Ne value of silica glass is significantly higher than that of polymer membranes, 150 at 400 °C [34], but the helium permeability is insufficient for industrial use [32,34]. A low-density silica structure (expanded silica glass) can have a higher helium permeability compared with ordinary silica glass and maintain a high selectivity for He-Ne mixture separation [35].

The gas transport properties of silicate glass depend on the composition and structure of the glass phase, which determine the gas migration in the interstitial space of the glass structural network formed by (SiO_4_)-tetrahedra. Modifier ions K^+^, Na^+^, Ba^2+^, Mg^2+^, Ca^2+^, etc., occupy interstitial voids in the structural network, thus preventing gas penetration [36,37]. In aluminosilicate glasses, aluminum cations, depending on the coordination, can act as glass formers (four- and five-coordinated) or modifiers (six-coordinated); after annealing at high temperatures, the six-fold coordination of aluminum becomes predominant, and Al-cations are randomly located in the space between silicon and oxygen tetrahedra [38,39,40]. The structure of aluminosilicate glass, when aluminum acts as a glass former, consists of separate Si-O and Al-O subnetworks. This different ordering of Al and Si atoms leads to microphase separation in the glass, causing the Al-O-rich network to leak through the Si-O network in a peculiar manner. The interweaving of networks leads to the formation of Al-rich areas, which serve as nucleation channels for the subsequent formation of needle-shaped crystallites of the mullite phase during crystallization [41,42]. Thus, crystallization in silicate and aluminosilicate glasses can have a significant effect on permeability since it promotes the displacement of modifier ions from the glass phase and changes the material structure.

There are practically no systematic data on the gas transport properties of nonporous silicate glass–crystalline composites in the literature. Investigating the relationship between the composition, structure, and gas permeability of these composite materials is important, especially when considering their potential applications as separation membranes. Such studies will fill the knowledge gap in the field of membrane materials science.

Coal fly ash cenospheres are promising for studying the gas transport properties of glass–crystalline materials in a wide range of compositions [20,21]. It was shown that the helium permeability of the glass phase of cenospheres significantly exceeds the analogous values for silicate glasses; in terms of density, the glass phase of cenospheres containing 10 mol % of modifier ions corresponds to silica glass, in which there are no modifier ions [21]. These studies concerned morphologically homogeneous narrow fractions of cenospheres containing single-ring-structure globules. Another morphological type of cenosphere is foamy globules with a network structure [20,43]; their diffusion properties relative to He and Ne have not yet been considered. The development of new membrane materials for the separation and purification of He and Ne based on microspherical components of fly ash from the industrial pulverized combustion of coal is a very urgent task. Solving this problem involves establishing the relationship “composition—structure—properties” for cenospheres of different types and identifying patterns of the formation of new functional materials with specific properties based on technogenic raw materials.

This paper is devoted to the characterization of silicate glass–mullite (SiG/M) composites based on narrow fractions of fly ash cenospheres with different globule structures as effective gas separation membranes. The research included the production of composite membranes, the study of their composition and structure, and the determination of gas transport properties to He and Ne. The expected unique combination of high permeability and selectivity along with the strength of the glass–crystalline shell of cenospheres makes these materials promising for effective helium–neon mixture separation and the production of high-purity gases.

## 2. Materials and Methods

### 2.1. Preparation of SiG/M Composites

SiG/M composite membranes based on narrow fractions of fly ash cenospheres were prepared by applying a previously developed technique, including the separation of cenosphere concentrates into narrow fractions [17,18,19] and the high-temperature treatment of cenosphere narrow fractions [20].

Concentrates of fly ash cenospheres produced after the industrial pulverized combustion of coal were used as a feedstock for obtaining narrow fractions of cenospheres with the maximum content of globules with a single-ring or network structure. The separation of cenosphere concentrates into narrow fractions was performed according to the technological scheme [17,18,19] including stages of aerodynamic classification and magnetic and grain-size separation. To obtain homogeneous fractions of cenospheres with reproducible physical and chemical characteristics, a unique technological complex of equipment was used, including an ATP 50 centrifugal laboratory aerodynamic classifier and an ALPINE e200 LS air-sieve screening unit (Hosokawa Alpine, Augsburg, Germany). The separation of cenospheres by magnetic properties was carried out at a magnetic field strength of 10.55 KOe.

**Figure 1:**
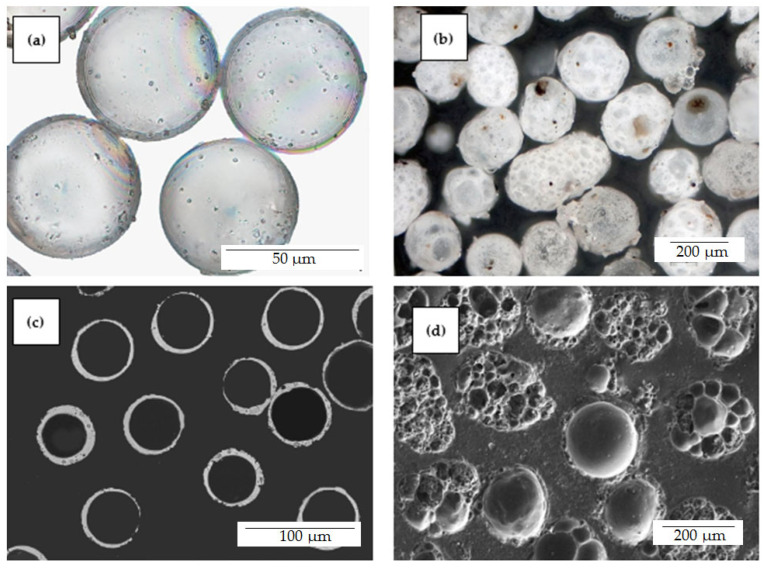
Optical images (**a**)—transmitted light, (**b**)—reflected light and SEM images of polished sections (**c**,**d**) of cenosphere narrow fractions. (**a**,**c**) M −0.063 + 0.05; (**b**,**d**) R −0.25 + 0.2.

**Figure 2:**
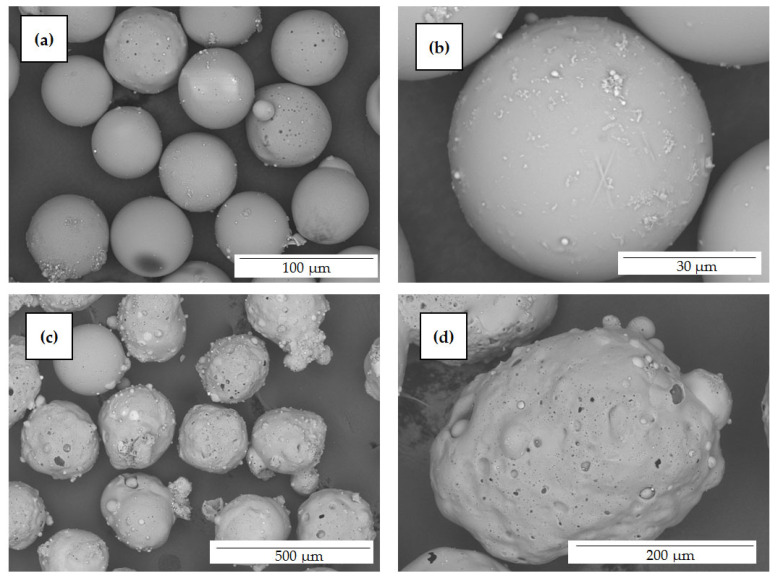
SEM images of cenosphere narrow fractions (**a,c**) and their individual globules (**b**,**d**). (**a**,**b**) M −0.063 + 0.05; (**c**,**d**) R −0.25 + 0.2.

As a result, a nonmagnetic narrow fraction with a size of −0.063 + 0.05 mm (series M) was isolated from the concentrate of fly ash cenospheres from the combustion of Kuznetsk coal at the Moscow Thermal Power Plant (flame kernel temperature of 1650 °C). From the concentrate of fly ash cenospheres after the combustion of Ekibastuz coal at the Reftinskaya TPP (flame kernel temperature of 1550 °C), a fraction −0.25 + 0.2 mm in size (series R) was obtained. The decisive factor in the choice of these fractions was the morphology of their globules. A cenosphere narrow fraction with a size of −0.063 + 0.05 mm is completely represented by spheres of a single-ring structure with a thin solid or low-porosity shell; a cenosphere narrow fraction with a size of −0.25 + 0.2 mm contains predominantly foamy particles with cavities of various sizes and globules with a highly porous shell (Figure 1). The chemical and phase compositions of the narrow fractions are summarized in Table 1 and Table 2; SEM images are shown in Figure 1c,d and Figure 2.

Narrow fractions of cenospheres M −0.063 + 0.05 and R −0.25 + 0.2 were subjected to heat treatment at 1000 and 1100 °C, respectively, in an oxidizing atmosphere for 3 h. This temperature regime was chosen according to the results of the thermal analysis (DSC-TG), which was performed using a Jupiter STA 449C synchronous thermal analysis unit (Netzsch, Selb, Germany). The crystallization temperatures of the phases were determined according to the observed specific exothermic effects. It was found that the phase crystallization occurred in the temperature range of 980–1100 °C. After the high-temperature treatment, the destroyed and thoroughly perforated globules were removed using the hydrostatic method with preliminary vacuuming.

### 2.2. Characterization of SiG/M Composites

For SiG/M composites, we determined the following physicochemical characteristics: bulk density, chemical and phase compositions, particle size distribution, average diameter of the globules, apparent thickness of the glass–crystalline shells, absolute density and composition of the glass phase, and content of the globules related in structure to a certain morphological type. The methods for determining these parameters are described in detail in [18,19,20,24].

The bulk density was measured using an automated Autotap density analyzer (Quantachrome Instruments, Boynton Beach, FL, USA) and the particle size distribution was measured using a MicroTec 22 laser particle sizer (Fritsch, GmbH, Idar-Oberstein, Germany).

The chemical composition, including the content of silicon, aluminum, iron, calcium, magnesium, and potassium oxides, was determined using chemical analysis according to the State Standard GOST 5382-2019 [44], which specifies the methods for component identification. The standard error of repeatability (S_n_) and the discrepancy between the results of the parallel determinations (R_max_) for each component, depending on its content, did not exceed what was accepted according to GOST 5382-2019.

**Figure 3:**
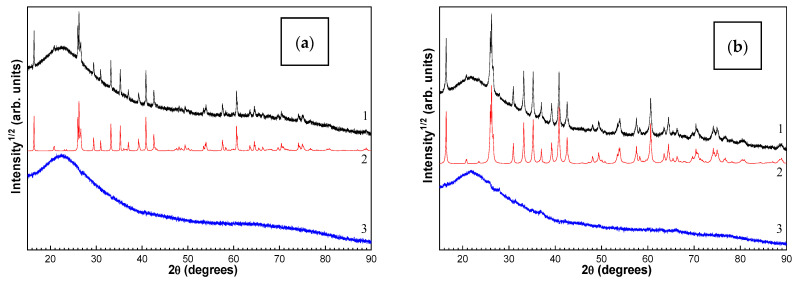

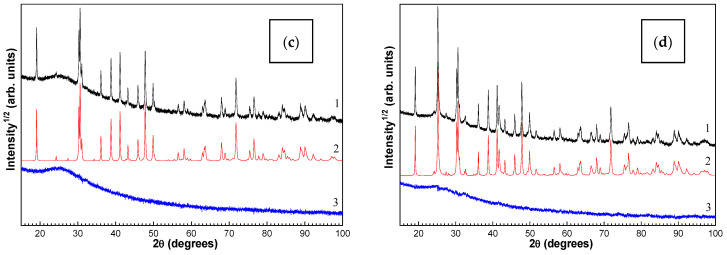
XRD patterns (1—experimental, 2—calculated, and 3—difference) for the initial narrow fractions of cenospheres (**a**,**c**) and SiG/M composites (**b**,**d**). (**a**) M −0.063 + 0.05; (**a**) M −0.063 + 0.05-1000; (**c**) R −0.25 + 0.2; and (**d**) R −0.25 + 0.2-1100.

The phase composition and structural parameters of the crystal phases (crystallite size and lattice parameters) were determined using quantitative X-ray powder diffraction analysis with the full-profile Rietveld method and the derivative difference minimization according to the procedure in [18]. The X-ray diffraction data were obtained on an X’Pert Pro MPD powder diffractometer (PANalytical, Almelo, The Netherlands) with a PIXcel solid-state detector using Cu Kα radiation (2θ range 12–120°). The weight percent of the X-ray amorphous component was determined through the external standard method with corundum used as the standard. The absorption coefficients of the samples were calculated from the total elemental composition according to the chemical analysis data. The composition of the glass phase was calculated using the set of data obtained through the chemical analysis and the quantitative X-ray phase analysis. Figure 3 shows the experimental and calculated XRD patterns and their difference from those for the original narrow fractions of cenospheres and the SiG/M composites obtained on their basis. The halo at 2θ~30–40° (line 3) indicates the presence of an X-ray amorphous phase in the sample.

The helium pycnometer method was used to determine the absolute density of the glass–crystalline shell of the cenospheres. The volume of a preliminarily ground cenosphere sample was measured using the difference in the helium pressure in a static volumetric measuring cell of an ASAP 2020C-MP (Micromeritics, Norcross, GA, USA) automated sorption analyzer.

The morphology of globules was studied using an Axioscop Imager D1 optical microscope equipped with an AxioCam MRc5 color digital camera (Zeiss, Carl-Zeiss-Stiftung, Oberkochen, Germany). The contents of globules of different morphological types were determined using the specially developed computer program “Msphere” for the processing of digital optical images of at least 5000 globules in each fraction [19].

The structure of the cenosphere shells was studied using TM-3000 and TM-4000 scanning electron microscopes (High Technologies Corporation, Hitachi, Tokyo, Japan). The composition of the single globules and their local areas were studied using the SEM-EDS method with a TM-3000 (SEM) equipped with a Quantax 70 microanalysis system and a Bruker XFlash 430H energy-dispersive X-ray spectrometer (EDS) (Bruker Corporation, Billerica, MA, USA) at a magnification of ×500−2500 and an accelerating voltage of 15 kV. Using polished sections of cenospheres in the elemental mapping mode, we analyzed the heterogeneity of the shells of individual globules. The data acquisition time was at least 10 min, which enabled the quantitative processing of the spectra. For single globules and their local areas, the gross composition, including the elemental content of Si, Al, Fe, Ca, Mg, Na, K, and Ti, was determined. The root mean square error (%) in determining the content of elements was O 1.4–3.1; Si 0.3–0.7; Al 0.2–0.6; Ca, Mg < 0.2; Na, K < 0.1; and Ti, Fe < 0.03. The elemental composition was converted to oxides and normalized to 100%.

### 2.3. Investigation of Gas Transport in SiG/M Composites

**Figure 4:**
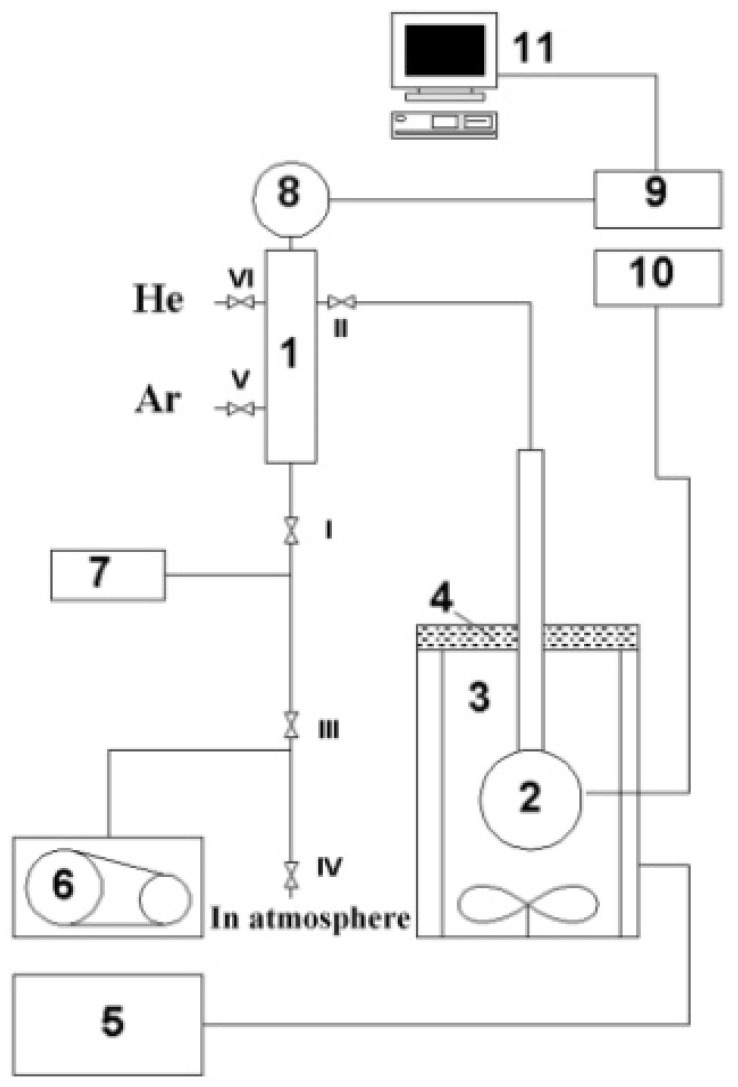
Schematic diagram of apparatus for measurements of pure gas (He, Ne) permeances: 1—manifold; 2—reactor; 3—gradientless furnace; 4—thermal insulator; 5—temperature controller RIF; 6—rotary oil vacuum pump DSE-II; 7—thermocouple vacuum sensor VIT; 8—pressure sensor AIR-20M; 9, 10—pressure and temperature converters; 11—computer; and I–VI—valves.

The pure gas (He, Ne) permeability of SiG/M composites was measured on a vacuum static installation (Figure 4) in the mode of gas diffusion from the reactor volume into the internal cavities of the cenospheres.

Gas transport through SiG/M composites is provided by the difference in partial pressures of the He or Ne outside and inside the globules. The measurements were carried out in the temperature range of 25–360 °C for helium and 280–500 °C for neon. The technique, including the determination of the He and Ne permeability coefficients, ideal αHe/Ne selectivity, and diffusion activation energy, is described in detail in [20]. The relative standard error of permeability determination did not exceed 10%.

## 3. Results and Discussion

### 3.1. Physical Characteristics and Chemical and Phase Composition of SiG/M Composites

**Figure 5:**
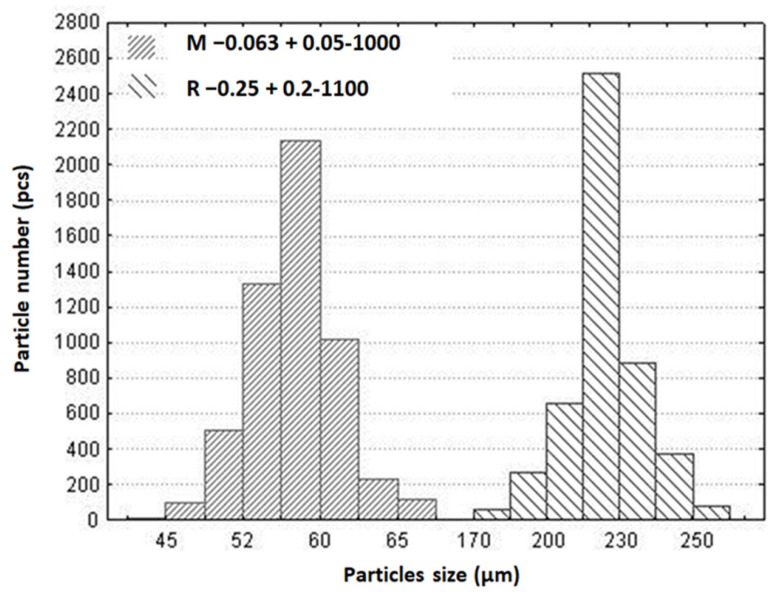
Particle size distribution of cenospheres in SiG/M composites.

SiG/M composites M −0.063 + 0.05-1000 and R −0.25 + 0.2-1100, prepared on the basis of cenosphere fractions with a size of −0.063 + 0.05 mm and −0.25 + 0.2 mm after heat treatment at 1000 and 1100 °C, respectively, are characterized by a narrow particle size distribution (Figure 5). The physical characteristics of SiG/M composites are presented in Table 3.

**Figure 6:**
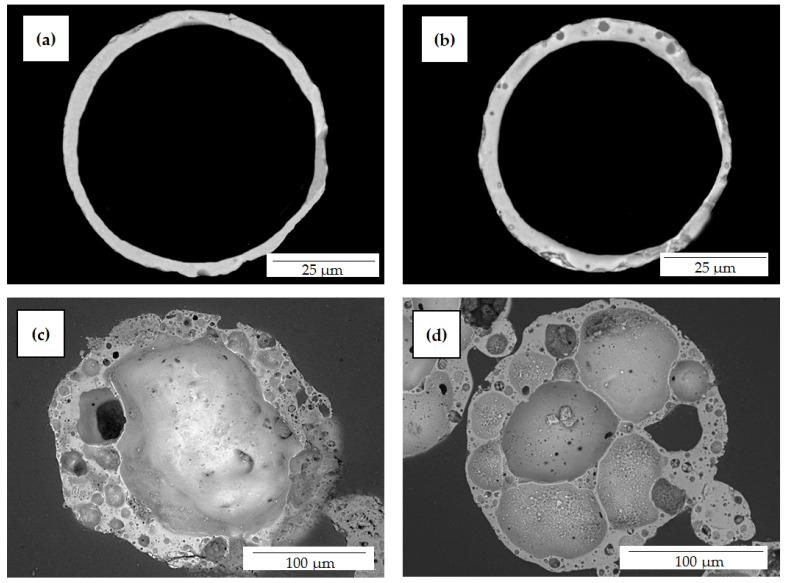
SEM images of polished shell sections of cenospheres with certain morphology in SiG/M composites (**a**,**c**) M −0.063 + 0.05-1000; (**b**,**d**) R −0.25 + 0.2-1100. (**a**) Single-ring structure with a thin solid shell; (**b**) single-ring structure with low-porosity shell; (**c**) highly porous, thick shell; and (**d**) network structure.

SiG/M composite M −0.063 + 0.05-1000 contains only single-ring structure spheres with a nonporous, thin, solid shell, 74%; with a low-porosity shell, 26%. In contrast, the composite SiG/M −0.25 + 0.2-1100 contains 57% globules with a typical network structure and 43% spheres with a highly porous, thick shell (Figure 6).

According to their chemical composition (Table 4), the composites are a multicomponent system with a total content of the main macrocomponents, SiO_2_ and Al_2_O_3_, of 93–95 wt%. The phase composition includes crystal phases and a glass phase (Table 5). Among the crystal phases, there are those that were present in the original cenospheres, mullite (0) and quartz. The mullite (I), β-cristobalite, and anorthite were formed after the high-temperature treatment of the cenospheres.

The structural characteristics of the main crystalline phases of SiG/M composites were determined. The study of the lattice parameters of mullite (I), quartz (I), and β-cristobalite revealed the imperfection of their crystal structure. Thus, the lattice parameters of mullite (I) are higher than those of mullite (0) (Table 6), which is associated with the formation of defective, iron-containing mullite [45,46,47]. The formation of mullite (I) in an oxygen atmosphere is accompanied by the oxidation of Fe^2+^ ions to Fe^3+^, their extraction from the glass phase, and their incorporation into the Al^3+^ ion positions of the crystallized mullite lattice [20,21]. For the quartz phase, two modifications with different lattice parameters were revealed (Table 5 and Table 7). One of them, quartz (0), is close to pure quartz (a = 4.91344 Å, c = 5.405–5.40524 Å) [48]; the other, quartz (I), is characterized by larger lattice parameters associated with the introduction of Al ions, which occurs due to the dissolution of quartz in the molten glass phase during the formation of cenospheres [18,49].

The lattice parameter of cristobalite is a = 7.1199(8) Å. The revealed cubic lattice corresponds to the high-temperature modification of β-cristobalite, the stabilization of which can be associated with the defectiveness of its structure. It is the structural defect associated with the introduction of aluminum ions, which is the reason for the stabilization of the high-temperature form of cristobalite.

The crystallite size of the mullite (0) phase in SiG/M composites is smaller than that of mullite (I) (Table 6). The sizes of quartz and β-cristobalite crystallites could not be determined due to the broadening of the peaks of these phases in the X-ray diffraction pattern. This may be due to microdistortions in the lattice due to the partial replacement of silicon ions by aluminum ions.

Based on the data from the chemical analysis and quantitative X-ray phase analysis (Table 4 and Table 5), the composition of the glass phase in the SiG/M composites was calculated (Table 8). The total content of oxide modifiers Na_2_O + K_2_O + CaO + MgO + Al_2_O_3_ in the silicate glass matrix is 17.5 wt% for M −0.063 + 0.05-1000 and half as much for R −0.25 + 0.2-1100—8.6 wt% (Table 8).

For comparison, the content of oxide modifiers in the glass phase of the original cenospheres is 27.8 wt% for the M −0.063 + 0.05 fraction and 19.6 wt%—for the R −0.25 + 0.2 fraction (Table 8). Table 8 shows the glass density values for the original cenospheres and composites based on them. It has been established that with a decrease in the content of modifier ions, the density of the glass phase decreases noticeably. This indicates that the glass phase of the SiG/M composites is characterized by an expanded structure formed due to the diffusion of modifier ions from the glass during the crystallization of defective phases in the cenosphere shells.

### 3.2. Structure and Composition of Individual Globules of SiG/M Composites

**Figure 7:**
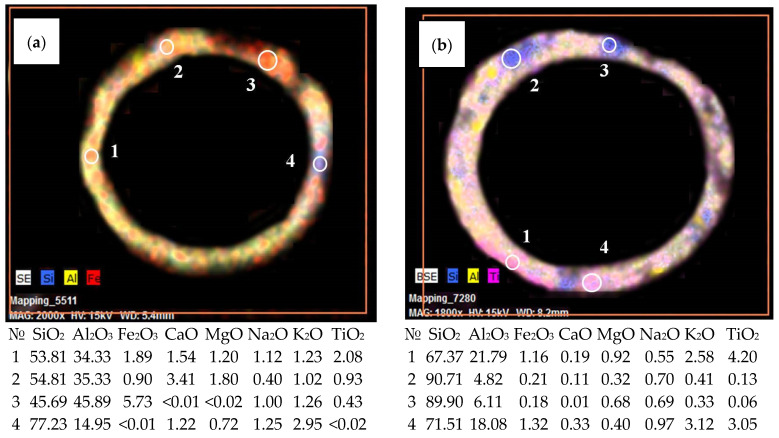

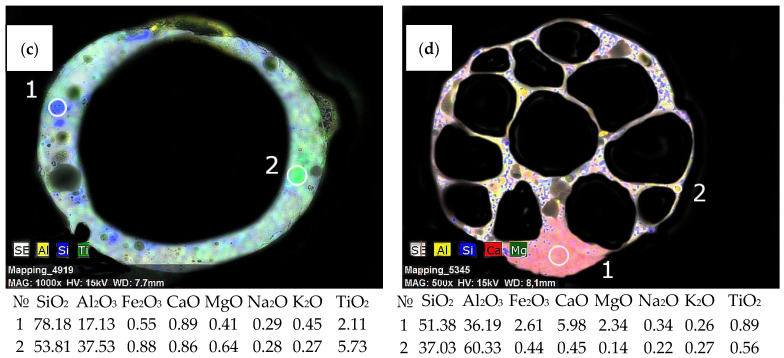
SEM images and distribution maps of some of the elements (highlighted in different colors) in polished shell sections of cenospheres in SiG/M composites (**a,b**) M −0.063 + 0.05-1000 and (**c,d**) R −0.25 + 0.2-1100, indicating heterogeneous areas and their compositions. (**a**) Single-ring structure with a thin, solid shell; (**b**) single-ring structure with low-porosity shell; (**c**) highly porous, thick shell; and (**d**) network structure.

The study of the gross composition of individual cenospheres using the SEM-EDS method showed that the SiG/M composites are characterized by a wide range of component contents (Table 9). Thus, for the M −0.063 + 0.05-1000 composite, the contents of the macrocomponents are 22–42 wt% of Al_2_O_3_ and 45–70 wt% of SiO_2_ (Table 9). The glass–crystalline shell is thin and monolithic, and it irregularly contains small, single pores (Figure 7a,b).

The ranges of the macrocomponent contents of individual cenospheres in the R −0.25 + 0.2-1100 composite are 24–50 wt% of Al_2_O_3_ and 38–63 wt% of SiO_2_ for globules with a single-ring structure, and 42–51 wt% of Al_2_O_3_ and 46–53 wt% of SiO_2_ for globules with a network structure (Table 9). With an increase in the content of aluminum oxide, we observed a monotonic change in the structure of the shell and a transition from ring-structure globules to spheres with large cavities, and then to network structure globules (Figure 7d).

**Figure 8:**
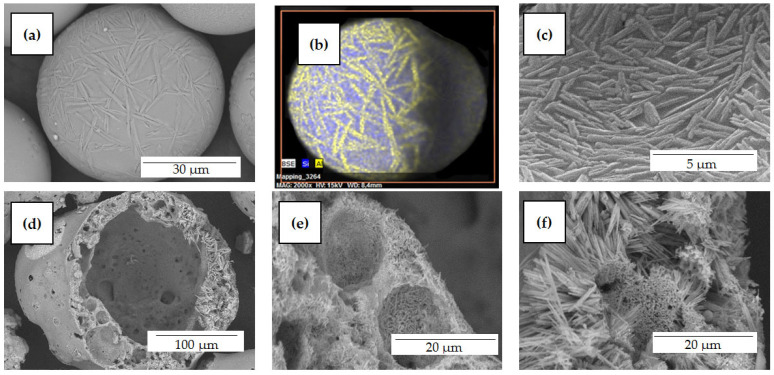
SEM images of cenosphere shell with mullite crystallites in SiG/M composites: (**a**–**c**) M −0.063 + 0.05; (**d**–**f**) R −0.25 + 0.2.

It has been established that individual cenospheres with a solid and porous shell, as well as those with a network shell (Figure 7), have a fragmentary structure, are heterogeneous in chemical composition, and contain areas with a high content of silicon, aluminum, iron, calcium, magnesium, and titanium. In the case of cenospheres with a single-ring structure, acicular mullite crystallites are formed on the outer (Figure 8a,b) and inner surfaces (Figure 8c) of the globules; for cenospheres of a network structure, volumetric crystallization of the shell is observed (Figure 8d–f). Microcrystalline mullite forms a kind of crystalline framework, which gives the cenospheres structural stability. The strength characteristics of network structure cenospheres are 3–5 times higher compared with ring-structure cenospheres.

### 3.3. Gas Transport Properties of SiG/M Composites

Glass–crystalline membranes can have improved gas transport properties due to the purification of the glass phase from the modifier ions [36,37] and the formation of an expanded low-density microstructure [35] during crystallization [21]. It was of interest to determine how the observed differences in the composition and structure of cenospheres would affect the gas permeability and selectivity of glass–crystalline materials obtained from them.

**Figure 9:**
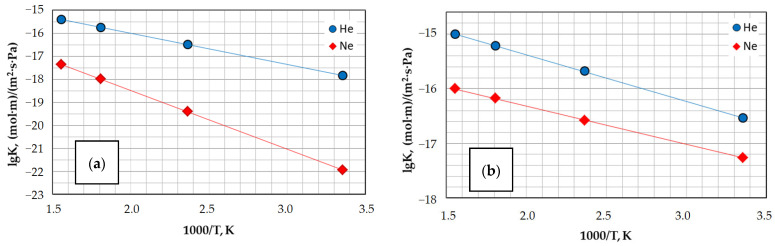
Temperature dependence of the He and Ne permeability coefficients for the glass phase of the SiG/M composites: (**a**) M −0.063 + 0.05-1000; (**b**) R −0.25 + 0.2-1100.

Figure 9 shows the temperature dependences of permeability coefficients He and Ne for the glass phase of SiG/M composites based on narrow fractions of fly ash cenospheres with different globule morphologies. It can be seen that over the entire temperature range, the composite R −0.25 + 0.2-1100, containing cenospheres of a network structure with a lower content of modifier ions in the glass phase, has higher permeability coefficient (K) values for the studied gases than the composite M −0.063 + 0.05-1000 with a single-ring globule structure and twice the content of modifier ions in the glass phase. If we compare the K values for these samples at 280 °C, then the observed excess will be almost an order of magnitude for helium and 1.5 orders of magnitude relative to neon (Table 10). The permeability coefficients of the glass phase of SiG/M composites exceed similar values for the initial cenospheres, which contain more modifier ions in the glass phase with a higher density, by 3–5 times for the test gases (Table 10). With a decrease in the content of modifier ions in the glass phase of cenospheres and SiG/M composites, the activation energies of the He and Ne diffusion processes decrease (Table 10).

A comparative analysis of the gas permeability of SiG/M composites and synthetic silicate glasses [34,50,51,52] at 280 °C was carried out (Table 11). The helium permeability coefficient for the R −0.25 + 0.2-1100 composite is 10 times higher than that of Corning Glass #7056 with the same modifier oxide content of 10 mol % and is equal to silica glass without modifiers.

The observed excess of K values of the SiG/M composite compared with silicate glass is associated with the fragmented structure of the cenosphere shells, containing areas enriched in SiO_2_ without modifier oxides (Figure 7), and the expanded microstructure of low-density glass, formed due to the crystallization of defective phases, mainly iron-containing mullite, cristobalite, and quartz with embedded aluminum cations. Lower activation energy values for the SiG/M composites (Table 10) compared with the silica glass (He—21 kJ/mol; Ne—38 kJ/mol [34]) indicate a facilitated gas diffusion process.

The ability of membranes to separate a mixture of two gases is generally defined as selectivity. For SiG/M composites, the ideal selectivity for αHe/Ne, which is the ratio of pure gas permeability (Table 10), is significantly higher than that for polysulfone [31], and it corresponds to silicate glass [34,51,52].

Thus, it has been established that the gas permeability of SiG/M composite membranes based on cenospheres is determined by the fragmentary structure of the glass–crystalline cenosphere shells, the presence of regions enriched with the glass-forming oxide SiO_2_ and not containing modifier ions, and the formation of an expanded glass during the crystallization process. Such compositional and structural features facilitate the process of gas diffusion compared with synthetic silicate glasses. A significant advantage of glass–crystalline membranes is the combination of high selectivity and permeability, which makes them promising for use in membrane separation and gas purification processes.

## 4. Conclusions

This work reports new silicate glass–mullite composites produced from narrow fractions of fly ash cenospheres with a size of −0.063 + 0.05 mm and −0.25 + 0.2 mm after heat treatment at 1000 and 1100 °C, respectively. The resulting composites are characterized by a predominant content of globules with a single-ring or network structure; they consist of a silicate glass matrix and defective phases of mullite, quartz, cristobalite, and anorthite. The SEM-EDS study showed that the cenosphere shells, regardless of their globules’ morphology, have a fragmentary structure, are heterogeneous in chemical composition, and contain areas enriched in SiO_2_ without modifier oxides. Needle-shaped crystallites of mullite are formed on the outer and inner surfaces of globules with a single-ring structure; for cenospheres with a network structure, the volumetric crystallization of the shell is observed, which gives them increased strength. The promising performance of silicate glass–mullite composites based on coal fly ash cenospheres for effective helium–neon mixture separation was demonstrated. The permeability coefficients He and Ne exceed similar values for silicate glasses; the selectivity corresponds to a high level, αHe/Ne 22 and 174 at 280 °C, which is significantly higher than that for polymer membrane materials. The results obtained can be used in the development of new, highly selective membrane materials with improved microstructure and gas transport characteristics for energy-efficient membrane technology for separating helium, hydrogen, and neon from gas mixtures, and for purifying helium concentrate from impurities.

## Figures and Tables

**Table 1:** Chemical composition of cenosphere narrow fractions (wt%).

Fraction	LOI	SiO_2_	Al_2_O_3_	Fe_2_O_3_	CaO	MgO	SO_3_	Na_2_O	K_2_O	P_2_O_5_
M −0.063 + 0.05	0.82	58.26	32.08	2.80	1.27	1.15	0.27	0.72	2.39	0.08
R −0.25 + 0.2	0.39	56.18	38.08	1.68	1.62	1.17	0.28	0.29	0.39	0.05

**Table 2:** Phase composition of cenosphere narrow fractions (wt%).

Fraction	Glass Phase	Mullite	Quartz	Calcite
M −0.063 + 0.05	89.4	8.4	1.5	0.7
R −0.25 + 0.2	61.5	36.7	1.7	0.1

**Table 3:** Physical characteristics and content of cenospheres with certain morphology in SiG/M composites.

Sample	Physical Characteristics				Content of Cenospheres (%)		
	Bulk Density (g/cm^3^)	Absolute Density (g/cm^3^)	Apparent Thickness of Shell (μm)	Average Diameter (μm)	Single-Ring Structure with Solid Shell	Single-Ring Structure with Porous Shell	Network Structure
M −0.063 + 0.05-1000	0.34	2.57	2.5	60	74	26	0
R −0.25 + 0.2-1100	0.43	2.67	11.3	227	0	43	57

**Table 4:** Chemical composition of SiG/M composites (wt%).

Sample	SiO_2_	Al_2_O_3_	Fe_2_O_3_	CaO	MgO	SO_3_	Na_2_O	K_2_O
M −0.063 + 0.05-1000	60.22	32.56	1.9	0.99	1.30	0.09	0.60	2.42
R −0.25 + 0.2-1100	59.30	35.66	1.54	1.00	0.71	–	0.32	0.40

**Table 5:** Phase composition of SiG/M composites (wt%) *.

Sample	Glass Phase	Mullite (0)	Mullite (I)	Quartz (0)	Quartz (I)	β-Crystobalite	Anortite
M −0.063 + 0.05-1000	73.7	7.2	16.5	0.5	2.1	–	–
R −0.25 + 0.2-1100	33.5	42.3	4.1	1.2	1.0	16.0	1.9

* (0)—designation of crystal phases present in the initial cenospheres; (I)—designation of the same phases formed in SiG/M composites after heat treatment of cenospheres.

**Table 6:** Lattice parameters of mullite in SiG/M composites.

Sample	Mullite (0)				Mullite (I)			
	a (Å)	b (Å)	c (Å)	Crystallite Size (µm)	a (Å)	b (Å)	c (Å)	Crystallite Size (µm)
M −0.063 + 0.05-1000	7.5655 (3)	7.6894 (2)	2.8871 (1)	143	7.572 (1)	7.7131 (9)	2.8929 (2)	24
R −0.25 + 0.2-1100	7.5579 (3)	7.6922 (3)	2.8875 (1)	142	7.564 (4)	7.728 (4)	2.898 (1)	82

**Table 7:** Lattice parameters of quartz in SiG/M composites.

Sample	Quartz (0)		Quartz (I) (Al, Si)O_2_	
	a (Å)	b (Å)	a (Å)	b (Å)
M −0.063 + 0.05-1000	4.906 (2)	5.420 (3)	4.940 (4)	5.422 (6)
R −0.25 + 0.2-1100	4.922 (2)	5.410 (4)	4.957 (4)	5.430 (6)

**Table 8:** Absolute density and chemical composition of the glass phase in the initial cenospheres and the SiG/M composites.

Sample	Absolute Density (g/cm^3^)	Chemical Composition (mol %)						
		SiO_2_	Al_2_O_3_	Fe_2_O_3_	CaO	MgO	Na_2_O	K_2_O
M −0.063 + 0.05	2.44	72.16	20.00	1.39	1.24	2.28	0.92	2.01
M −0.063 + 0.05-1000	2.38	82.54	9.16	–	1.71	3.15	0.94	2.50
R −0.25 + 0.2	2.43	80.40	11.37	1.12	2.99	3.13	0.50	0.44
R −0.25 + 0.2-1100	2.37	91.45	1.12	–	2.15	3.45	1.00	0.83

**Table 9:** Minimum and maximum values of the oxide content (wt%) in individual cenospheres with certain morphology.

	SiO_2_	Al_2_O_3_	Fe_2_O_3_	CaO	MgO	Na_2_O	K_2_O	TiO_2_	MnO
M −0.063 + 0.05-1000; single-ring structure									
Min	45.18	21.74	0.82	0.24	0.46	0.63	0.81	<0.01	<0.01
Max	69.51	41.74	3.76	3.60	2.16	7.70	9.17	1.15	0.22
R −0.25 + 0.2-1100; single-ring structure									
Min	37.56	23.83	0.65	0.38	0.24	0.38	0.44	0.64	<0.01
Max	62.94	49.28	7.78	3.17	2.18	1.10	2.25	1.99	0.13
R −0.25 + 0.2-1100; network structure									
Min	45.89	41.28	0.50	0.39	<0.01	<0.01	0.06	0.33	<0.01
Max	53.41	50.51	1.08	1.89	0.49	0.83	1.44	4.12	0.06

**Table 10:** Gas transport properties of the initial cenospheres and SiG/M composites.

Sample	Content of Modifier Oxide in Glass Phase (mol %)	K (mol·m)/(m^2^·s·Pa) at 280 °C		αHe/Ne at 280 °C	Activation Energy (kJ/mol)	
		He	Ne		He	Ne
M −0.063 + 0.05	27.84	6.68·10^−17^	3.18·10^−19^	210	30	43
M −0.063 + 0.05-1000	17.46	1.77·10^−16^	1.02·10^−18^	174	26	39
R −0.25 + 0.2	19.55	4.91·10^−16^	1.46·10^−17^	34	19	21
R −0.25 + 0.2-1100	8.55	1.47·10^−15^	6.66·10^−17^	22	16	13

**Table 11:** Gas transport properties of silicate glasses at 280 °C.

Sample	Content of Modifier Oxide (mol %)	He Permeability Coefficients (mol·m)/(m^2^·s·Pa)	References
Corning Glass #7056	10	1.1·10^−16^	[50]
Vicor glass	4	6.7·10^−16^	[52]
Silica glass	0	1.3·10^−15^	[34,51]

## Data Availability

Not applicable.
